# Autonomic neurosurgery: from microvascular decompression to image guided stimulation

**DOI:** 10.2349/biij.3.1.e14

**Published:** 2007-01-01

**Authors:** EAC Pereira, AL Green

**Affiliations:** Oxford Functional Neurosurgery, Department of Neurosurgery, Radcliffe Infirmary, United Kingdom

**Keywords:** Autonomic, deep brain stimulation, microvascular decompression, neurosurgery, periaqueductal grey, rostral ventrolateral medulla

## Abstract

The paper reviews mechanisms underlying autonomic disorders, with a focus on cardiovascular dysfunction. Neurosurgical approaches are described for medically refractory hypertension and orthostatic hypotension. After review of microvascular decompression of the rostral ventrolateral medulla, stereotactic CT and MRI guided deep brain stimulation of the periaqueductal grey matter (PAG) is evaluated. Results are presented from patient studies showing reductions in blood pressure with ventral PAG stimulation and increases in blood pressure with dorsal PAG stimulation. A rationale for the treatment of autonomic disorders by neurosurgical intervention is discussed.

## INTRODUCTION

The autonomic nervous system comprises segregated and differentially regulated neuronal pathways linking the central nervous system with endocrine glands, smooth muscle and visceral organs via central neuron groups and peripheral ganglia [1, 2]. Disorders of autonomic function give rise to cardiovascular, gastrointestinal, genitourinary and endocrine disturbances [3]. Such disturbances are often poorly understood and managed, one reason being that autonomic dysfunction spans several medical specialties and requires a strong conceptual understanding of physiology and anatomy. The interpretation of autonomic investigations is frequently challenging.

Of the autonomic disorders, alongside the neuropathic complications of diabetes mellitus, the cardiovascular disorders of hypertension and orthostatic hypotension refractory to medical treatment present a considerable disease burden with high associated morbidity and mortality. Up to 27% of hypertensive people have poorly controlled blood pressure [4]. 3% are refractory to pharmacotherapy [5], and are therefore at considerably increased risk of both cerebrovascular and cardiovascular adverse events [6, 7]. A wealth of animal literature depicts brainstem regions in cardiovascular function ranging from demonstrations of neurogenic shock secondary to infrapontine transection by Dittmar in the 1870s and the later elucidation of its sympathetic vasomotor component [8]. In particular the rostral ventral medulla, adjacent vagus and periaqueductal grey matter are potential targets for therapy for such autonomic disorders. Advances made towards understanding these autonomic disorders and their treatment using neurosurgical interventions and imaging adjuncts are described here.

## MICROVASCULAR DECOMPRESSION OF THE ROSTRAL VENTROLATERAL MEDULLA

A rationale for intracranial intervention to treat medically refractory hypertension developed from the hypothesis that exerting pressure upon cranial nerves by ectatic perimesencephalic vasculature could result in cranial nerve palsies. Cranial nerve dysfunction could be ameliorated by retromastoid craniectomy and microvascular decompression of the posterior fossa [9, 10]. Contentious at first [11], the procedure became established in the past two decades as the surgical therapy of choice for trigeminal neuralgia and for the relief of hemifacial spasm and glossopharyngeal neuralgia [12]. Many patients hypertensive prior to surgery exhibited vascular compression of the rostral ventrolateral medulla (RVLM) and adjacent dorsal glossopharyngeal and vagal nerve roots, with subsequent normotension achieved in several patients in whom such compression was relieved [13, 14].

Neurosurgery has been transformed by the introduction of the operating microscope and endoscopes have proven advantageous in minimizing the invasiveness of microvascular decompression [15, 16]. Moreover, advances in imaging have contributed to the clinical assessment of symptomatology suggestive of putative perimesencephalic neurovascular compression syndromes. Magnetic resonance angiography (MRA) often detects a problem [17-19], with high resolution magnetic resonance imaging (MRI) methodologies promising to advance diagnosis [20-22]. However, current MRI technology remains unable to consistently detect compression amenable to surgery [23-25].

The results of microvascular decompression of the glossopharyngeal and vagal cranial nerves for medically refractory hypertension suggest that one-third to one-half of patients show a sustained, long-term improvement [26, 27]. Neurosurgery for hypertension is predicated first if the hypertension is severe and medically refractory and second if the hypertension is not due to renal or endocrine abnormalities such as phaeochromocytoma, that is to say ‘essential’ in nature. The corollary therefore is that essential hypertension is neurogenic in origin.

The theoretical argument for microvascular decompression is that the RVLM is not only a centre for cardiovascular control, but vagal afferents from the myocardium to the nucleus of the solitary tract pass preferentially along the left aspect of the RVLM and therefore the sympathetic tone and cardiac contractility are particularly sensitive to prolonged pulsatile compression of this region. The argument is borne out by elegant animal models [28-30] and other contributors to hypertension can be explained within the model: for example, age and atherosclerosis are causes of vascular ectasia with reduced mural compliance. However the results of the small surgical case series conducted thus far remain equivocal. Larger controlled trials with confirmatory pre- and post-operative imaging and post-mortem evidence are required to confirm both the benefits and mechanism.

## DEEP BRAIN STIMULATION OF THE PERIAQUEDUCTAL GREY MATTER

The periaqueductal grey area (PAG) is established as a region important to the modulation of pain. It has been targeted by deep brain stimulating electrodes for the treatment of chronic, intractable neuropathic pain for three decades [31-34]. In mammals, the region is instrumental in ‘defence’ reactions [35], integrating descending responses from forebrain to cardiovascular effector organs to assist survival by modulating active sympathetic ‘fight’and ‘flight’ or passive ‘fear’ and ‘withdrawal’ responses [36-38]. Whether the coping is active or passive seems to determine whether the sympathetic response is pressor or hypotensive and the concomitant analgesia opioid or non-opioid mediated in animals [39]. Electrical stimulation of the PAG in animals elicits such defence reactions, dorsal regions being associated with active coping and hypertensive effects and ventral regions with passive coping and hypotensive effects [40-42]. Thus, it is likely that stimulation of the same area in the human will affect not only pain modulation pathways, but also other autonomic pathways including those affecting cardiovascular function. The PAG receives ascending projections from the RVLM [43], and much animal evidence implicates it in cardiovascular control [44, 45], pioneering demonstrations having been conducted in felines [46]. Additionally, the neurosurgical treatment of deep brain stimulation for pain enables the study of such cardiovascular effects in humans.

The technique of deep brain stimulation is an image-guided stereotactic neurosurgical intervention. Techniques vary between neurosurgical centres. Our technique is briefly outlined here. After a pre-operative T-1 weighted MRI scan, a Cosman-Roberts-Wells base ring is applied to the patient’s head under local anaesthesia. A stereotactic computerised tomography (CT) scan is then performed and the MRI scan is volumetrically fused to it using Radionics Image Fusion^®^ and Stereoplan^®^ programs to eliminate spatial distortions that arise from magnetic field effects [47, 48]. The co-ordinates for the PAG and entry trajectory are then calculated. A frontal trajectory avoiding the lateral ventricles is preferred. Targets are contralateral to the painful side of the body. After a parasagittal scalp incision and separate 2.7 mm twist drill craniotomy, the PAG is implanted with a Medtronic™ 3387 quadripolar electrode. The electrode is fixed to the skull by a miniplate and it is externalised parietally via temporary extensions. After surgery, a further stereotactic CT is performed and co-registered as before to confirm electrode position. After a week of post-operative assessment, a decision is made whether to permanently implant the electrode in a second operation under general anaesthesia. It is connected to a pulse generator implanted subcutaneously, usually infraclavicularly in subcutaneous facia. Our surgical technique is detailed further elsewhere [33, 49, 50].

Preliminary evidence has associated efficacious PAG analgesia using deep brain stimulation in humans with hypertensive and chronotropic cardiovascular effects [51, 52]. Our recent research has delineated in greater detail the cardiovascular effects of PAG deep brain stimulation [53-55]. The results are summarised here.

## ALTERING BLOOD PRESSURE BY DEEP BRAIN STIMULATION

**Figure 1:**
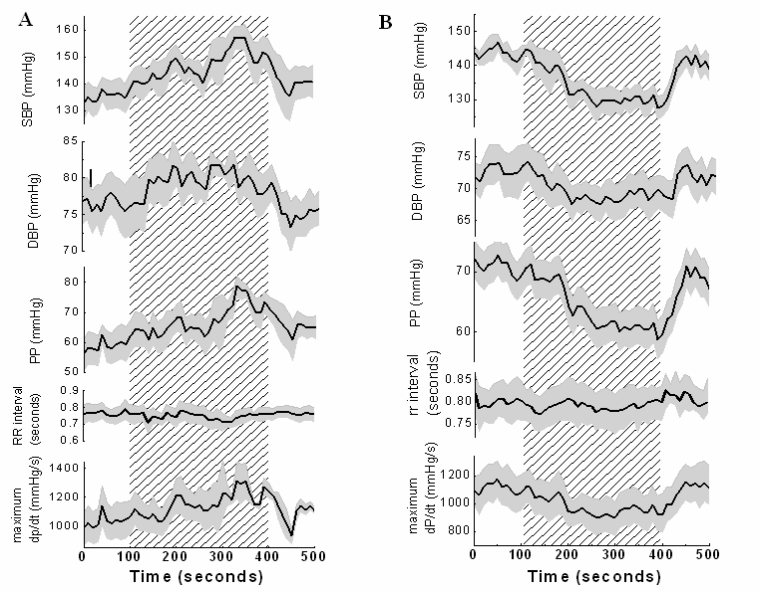
(a) Changes in cardiovascular parameters associated with reduced blood pressure. Patterned area = period of stimulation. Grey area = ± one standard error of the mean. SBP= systolic blood pressure, DBP= diastolic blood pressure. PP= pulse pressure, RR interval= time period between R waves on electrocardiogram, dP/dt = change of systolic blood pressure with time. (b) Changes in cardiovascular parameters associated with increased blood pressure. (see text for details).

We have shown that electrical stimulation of the human PAG alters blood pressure [55]. In this study of fifteen chronic neuropathic pain patients (17 electrodes), blood pressure and heart rate (via electrocardiogram) were continuously measured in the laboratory while deep brain stimulation parameters were altered from 10 Hz to 50 Hz (analgesic frequencies) [50]. We found that cardiovascular responses to stimulation were consistent, as measured on at least three occasions, for any pair of electrode contacts used. Arterial blood pressure reduced significantly overall in seven pairs of electrode contacts in seven patients. Conversely, blood pressure increased significantly in six pairs of contacts in six patients (p<0.05, ANOVA; Figure 1).

For the subjects with reductions in blood pressure, the average reduction was 14.2±3.6 mmHg (range 7-25mmHg), or 13.9%, after 300 seconds stimulation. Figure 1a shows the drop in systolic blood pressure (SBP) accompanied by a fall in diastolic BP (DBP) of 4.9mmHg ± 2.9 (p=0.03, single factor ANOVA, n=7, range 1.5-9.3), equivalent to 6%. This implicates a vasodilatatory mechanism. However, the greater systolic reduction with consequently reduced pulse pressure suggests additional central cardiovascular influences. We therefore measured the change of SBP with time (maximum dP/dt, i.e., the gradient of the blood pressure curve), a known marker of cardiac contractility [56]. The measures revealed a mean reduction of 222 mmHg/s ±126 (19.8%, p=0.06), suggestive of reduced myocardial contractility. In contrast, the R-R interval, a measure of heart rate, remained largely unchanged throughout the stimulation period (mean change = 0.01s ±0.04, range 0-0.08). As heart rate is controlled via the vagus, this implies no vagally mediated parasympathetic changes.

For those subjects experiencing increased blood pressure, the mean rise in SBP was 16.7 mmHg ±5.9 (p<0.001, single factor ANOVA, n=6, range 16-31mmHg), equivalent to 16.4% at the end of a 400s period where stimulation was started at 100s. Identical stimulation parameters of frequency 10 Hz, pulse width 120 μs and up to 3V amplitude both raised and lowered blood pressure in different contact pairs. As with blood pressure reduction, increases were accompanied by a smaller rise in DBP of 4.9mmHg ±2.8 or 6.4% (p=0.04, single factor ANOVA, n=6, range = 2.4 to 12.1mmHg). An increase in mean pulse pressure was also observed. Maximum dP/dt increased by 212±97 mmHg/s (p<0.03, single factor ANOVA). As for blood pressure reduction, the R-R interval did not change changed. Thus pressor and hypotensive related cardiac effects of stimulation appear to mirror each other.

Six control patients with implanted neurostimulatory interventions were investigated (six thalamic electrodes, one spinal cord stimulator). Despite extensive investigation using multifarious frequencies, pulse widths and voltages, and a variety of electrode contact configurations, we were unable to alter their blood pressure significantly. In addition to the control electrodes without cardiovascular effect, four patients with PAG electrodes (six electrodes in total) also failed to exhibit blood pressure changes.

**Figure 2:**
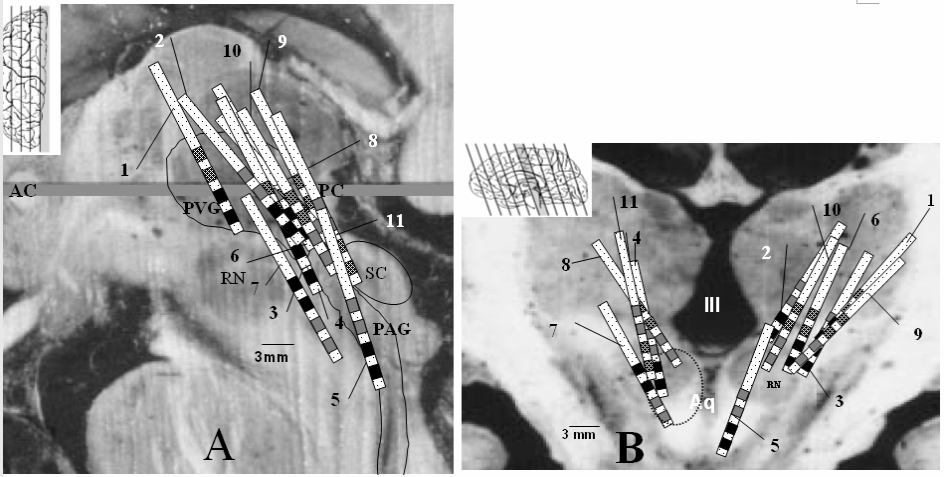
(a) Sagittal positions of the electrodes in patients in whom there were changes in blood pressure. (b) Coronal positions. For clarity, patients with no changes are not shown. Note that patient’s #1-7 all had reduction in BP (black contacts) and are the most ventral electrodes. Conversely, #8-11 and the upper 2 contacts of #1 and #6 had a rise in BP (patterned contacts). Gray contacts are those that, when stimulated, had no effect on BP. AC=anterior commissure, PC=posterior commissure, PVG=periventricular gray, PAG=periaqueductal gray, SC=superior colliculus (the level of which is depicted by the dotted circle in 1B), RN=red nucleus, III=third ventricle, Aq=aqueduct. Inset of A shows the ACPC plane, inset of B shows the slice position.

Because blood pressure changes in animals vary depending on whether the electrode is in ventral or dorsal PAG, electrode position was assessed. Electrodes were plotted on a brain atlas [57] using the post-operative MRI and a manipulation program (MRIcro version 1.38 build 1, Chris Rorden). Those electrodes that reduced blood pressure were placed ventrally and those that increased blood pressure dorsally (Figure 2). Of the patients without blood pressure changes, four of the five electrodes available for plotting were dorsal to the group that raised BP and hence probably beyond the PAG. The remaining electrode was in mid-periventricular grey.

Comparing changes in blood pressure between the two groups of patients with ventrally and dorsally situated electrodes (n=8 and n=9 respectively) including those without significant changes, the mean peak change in SBP was -10.3±2.8 mmHg for the ventral group and +10.8±3.1 mmHg for the dorsal group (p=0.003, one-way ANOVA). Similarly, the mean peak change in DBP was -4.6±1.2 mmHg and +3.5±0.8 mmHg respectively (p=0.007). Mean peak change in pulse pressure was -8.6±3.5 mmHg for the ventral and +7.4±2.1 mmHg for the dorsal group (p=0.01). dP/dt changes were -181.6±28 mmHg/s for ventral and 82±26 mmHg/s for dorsal electrode groups (p=0.007). Comparison of RR intervals between the two groups did not reveal significant differences (p=0.13).

**Figure 3:**
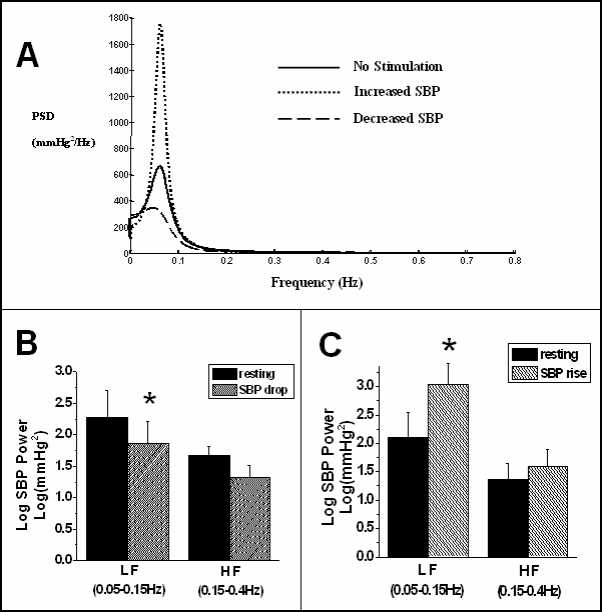
(a) Changes in low and high frequency power spectra of systolic blood pressure. A. Example in one patient, in whom blood pressure could be increased or decreased, depending on which contacts were used. A change in the low frequency component was associated with change in blood pressure, implying changes in sympathetic nervous system activity. (b,c) Changes for the groups in whom blood pressure decreased (N=7) or increased (N=6) respectively. Error bars denote ±one SEM.

Analysis of the dominant frequencies in the blood pressure wave form clarifies the underlying mechanisms of blood pressure changes [58]. Mayer’s wave (<0.1 Hz) suggests sympathetic autonomic control [59, 60]. Using autoregressive power spectral analysis [61] blood pressure changes were associated with alterations in sympathetic tone (Figure 3a). For group results, logarithms of the power of the low and high frequency components as the integral of the power spectra between 0.05 and 0.15Hz and between 0.15 and 0.4Hz were calculated for each of the increased and decreased blood pressure groups on and off stimulation with significance testing using a paired t-test (Figures 3c and 3d). The results showed significant low frequency power-spectra changes corresponding to blood pressure changes.

## FROM AUTONOMIC MICROVASCULAR NEUROSURGERY TO AUTONOMIC FUNCTIONAL NERUOSURGERY

We have demonstrated that it is possible to increase or decrease blood pressure in humans with electrical stimulation of the PAG. Furthermore, the direction of blood pressure change can be controlled by placing the electrode in either ventral or dorsal PAG. Reducing blood pressure with deep brain stimulation is theoretically possible but in itself poses a risk. Whereas DBS surgery entails a 0.3% stroke risk and is not inexpensive [62, 63], the changes shown augur well for its potential utility in the treatment of medically refractory hypertension. Further research requires demonstration of a sustained effect in addition to further elucidation of mechanisms of action. Moreover, for microvascular decompression which carries risks of cerebrospinal fluid leak (1.9%), deafness (0.8%) and cerebellar damage (0.4%) [25], the procedural risks need to be reduced before the procedure is applied to prophylaxis of hypertension rather than treatment of an established disability such as stroke [33, 64]. Future advances in stimulator technology may yield such risk reductions. The future potential of deep brain stimulators whose parameters adapt to control changing pain or degree of blood pressure abnormality also make such an intervention appealing [65, 66].

**Figure 4:**
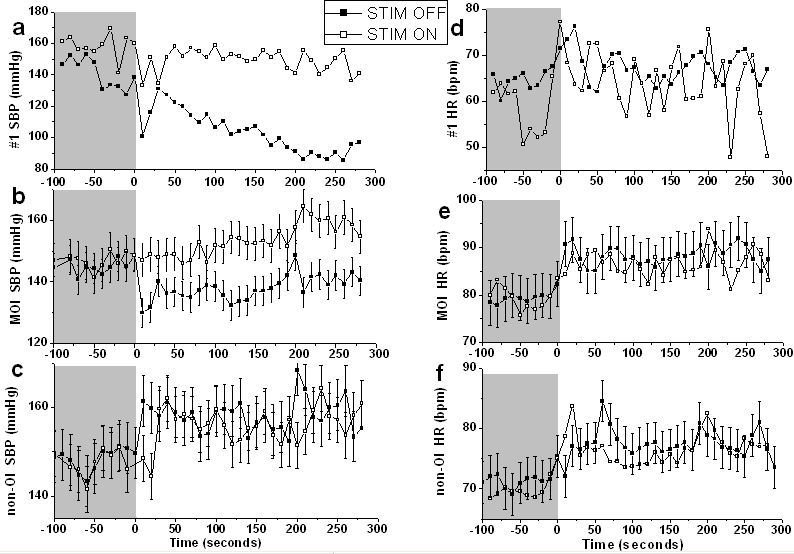
Blood Pressure and Heart Rate changes on standing. a-c: mean changes in systolic blood pressure for subject #1, MOI group, and non-OI group respectively. d-f: changes in heart rate for the same groups. All traces include the mean of three sessions, averaged every ten seconds. MOI= mild orthostatic intolerance group, non-OI = no orthostatic intolerance group. Grey area= period when patient was sitting, white area (from 0 seconds) = period of standing. ■ = stimulation ‘off’, □ = stimulation ‘on’. Error bars show ± one standard error of the mean.

The demonstration that PAG deep brain stimulation can increase and decrease blood pressure raises the possibility that orthostatic or postural hypotension might be treatable by neurosurgery. In the normal subject, assumption of an upright posture leads to pooling of venous blood in the lower extremities and splanchnic circulation. The resulting decrease in venous return to the heart leads to a compensatory, centrally mediated increase in sympathetic and decrease in parasympathetic activity (known as the baroreceptor reflex). Such activity normally causes a transient fall in SBP (5 to 10mmHg) a small rise in DBP (5 to 10mmHg) and a rise in heart rate of 10-25 beats per minute. In orthostatic hypotension, patients suffer troublesome low blood pressure on standing or symptoms of cerebral hypoperfusion [67]. Occuring in up to a fifth of people over 65 years of age, its treatment may lead to troublesome raised blood pressure [68, 69] . The presented evidence for pressor effects of dorsal PAG stimulation together with supportive animal experiments showing baroreflex vagal bradycardia inhibition with stimulation in rats [70] suggest that such stimulation could influence the baroreceptor reflex. Our human studies support such a theory [53]. Eleven patients including one with orthostatic hypotension resolved with PAG deep brain stimulation and five with mild orthostatic intolerance (MOI; a fall in systolic blood pressure of >20mmHg on standing, but no clinical symptoms) demonstrated significant reductions in postural drops in blood pressure (p<0.001, t-test; Figures 4a and 4b) compared to a control group without postural blood pressure changed (Figure 4c).

Assessing baroreflex sensitivity gives insight into the mechanisms underlying postural changes. In healthy subjects, baroreflex sensitivity decreases on standing [71, 72]. In autonomic neuropathy, such as that of diabetes, it has been shown that it is lower in the supine position with a diminished reduction on standing compared to normal subjects [72]. Calculating the baroreflex sensitivity index from the transfer function of systolic blood pressure and RR interval signals using bivariate autoregressive modelling [73], we showed that the baroreflex sensitivity in the subject with orthostatic hypotension and the group with MOI were similar to those with a mild autonomic neuropathy (table 1). We also showed that stimulation significantly raised sensitivity in the sitting position (t-test, p=0.018, <0.001 and 0.002 for subject #1, MOI and non-MOI groups respectively) and reduced the magnitude of reduction on standing in orthostatic hypotension (p=0.024 subject #1, p<0.001 MOI). This suggests that the postural blood pressure changes reversed by deep brain stimulation of the dorsal PAG act via a central mechanism to increase baroreceptor sensitivity.

**Table 1:** Changes in Heart Rate Variability and Baroreflex sensitivity while sitting and standing, with stimulation on or off. MOI= mild orthostatic intolerance. Ranges shown in brackets. *indicates significant difference between Off and On conditions within each group

Group:	**Subject #1**		**MOI group**		**Non-MOI group**	
Stimulation:	**OFF**	**ON**	**OFF**	**ON**	**OFF**	**ON**
**Low Frequency (ms2)**						
Sitting	5.2	4.7	69.8 (1-285)	136.2* (1-474)	208.1 (81-330)	466.3* (298-489)
Standing	1.6	4.9	7.0 (0.1-20)	135.0* (1-520)	313.5 (63-603)	346.9* (86-636)
**High Frequency (ms2)**						
Sitting	6.9	7.4	176.0 (3-730)	182.1 (1-757)	224.6 (10-618)	341.4 (9-727)
Standing	4.2	7.6	122.4 (1-600)	181.8* (1-751)	247.5 (6-521)	464.6* (5-1280)
**Baroreflex sensitivity index (ms/mmHg)**						
Sit	3.6	11.1	6.6 (2.5-10.7)	8.6* (7.2-11.1)	9.9 (2.9-17.3)	15.3* (6.9-23.8)
Stand	0.13	5.41	0.7 (0.1-2)	4.3* (0.5-9.2)	4.5 (0.1-8.3)	14.7* (4.2-26)

## CONCLUSIONS

Neurosurgery for the treatment of autonomic disorders has thus far embraced two approaches. The first, posterior fossa decompression of the left RVLM utilising microscopy and also endoscopy has shown some sustained benefit in small case series and has been vindicated to some extent by MRA findings of perimesencephalic vascular compression as a risk factor for hypertension [74]. However, further controlled studies in larger samples of patients are required to confirm its benefit, and its extension to the amelioration of acquired diabetes mellitus appears questionable and less robustly supported by radiological evidence [74, 75].

The second functional neurosurgical approach, utilising sterotaxy, CT, MRI and deep brain stimulation technology has demonstrated blood pressure changes in an intensively studied group of patients treated by electrodes in their midbrain PAG for chronic pain. Repeatable changes in blood pressure dependant upon whether an electrode is in ventral or dorsal PAG have been demonstrated, albeit over short periods of time. Theoretical foundations for the treatment of both essential hypertension and orthostatic hypotension have been derived from the results presented.

Both interventions are exciting not only because they translate research directly from animal models to humans but because of their vivid illustration of potential clinical therapies. Essential hypertension treated without drugs appeals because their adverse effects would be avoided. Similarly, drug treatment of orthostatic hypotension cannot differentiate between the supine and standing positions and may therefore lead to nocturnal hypertension [68, 69], a predicament potentially resolved by demand driven or posturally sensitive deep brain stimulators. A myriad of related and difficult to treat autonomic syndromes, for example orthostatic hypertension [76], may also respond to neural stimulation of central autonomic brain centres. Parallel advances in both the interventions themselves and the imaging modalities they utilise will enable greater understanding of autonomic dysfunction and lead to definitive and effective treatment. After these promising research-driven beginnings, autonomic neurosurgery should demonstrate long-term efficacy in larger samples of patients and favourable cost-benefit over lifelong medication before it becomes the treatment of choice for such disorders.

## References

[1] Blessing WW (1997). The lower brainstem and bodily homeostasis. Vol. xiv.

[2] Loewy AD, Spyer KM (1990). Central regulation of autonomic functions. Vol. xii.

[3] Mathias CJ, Bannister R (1999). Autonomic failure: a textbook of clinical disorders of the autonomic nervous system. 4th ed edition. Vol. xxix.

[4] McBride W, Ferrario C, Lyle PA (2003). Hypertension and medical informatics. J Natl Med Assoc.

[5] Alderman MH, Budner N, Cohen H (1988). Prevalence of drug resistant hypertension. Hypertension.

[6] Almgren T, Persson B, Wilhelmsen L (2005). Stroke and coronary heart disease in treated hypertension -- a prospective cohort study over three decades. J Intern Med.

[7] Wang JG, Staessen JA, Franklin SS (2005). Systolic and diastolic blood pressure lowering as determinants of cardiovascular outcome. Hypertension.

[8] Alexander RS (1946). Tonic and reflex functions of medullary sympathetic cardiovascular centers. J Neurophysiol.

[9] Jannetta PJ (1967). Arterial compression of the trigeminal nerve at the pons in patients with trigeminal neuralgia. J Neurosurg.

[10] Gardner WJ (1962). Concerning the mechanism of trigeminal neuralgia and hemifacial spasm. J Neurosurg.

[11] Adams CB (1989). Microvascular compression: an alternative view and hypothesis. J Neurosurg.

[12] McLaughlin MR, Jannetta PJ, Clyde BL (1999). Microvascular decompression of cranial nerves: lessons learned after 4400 operations. J Neurosurg.

[13] Jannetta PJ, Segal R, Wolfson SK (1985). Neurogenic hypertension: etiology and surgical treatment. I. Observations in 53 patients. Ann Surg.

[14] Jannetta PJ, Gendell HM (1979). Clinical observations on etiology of essential hypertension. Surg Forum.

[15] Rak R, Sekhar LN, Stimac D (2004). Endoscope-assisted microsurgery for microvascular compression syndromes. Neurosurgery.

[16] Abdeen K, Kato Y, Kiya N (2000). Neuroendoscopy in microvascular decompression for trigeminal neuralgia and hemifacial spasm: technical note. Neurol Res.

[17] Naraghi R, Geiger H, Crnac J (1994). Posterior fossa neurovascular anomalies in essential hypertension. Lancet.

[18] Kuncz A, Voros E, Barzo P (2006). Comparison of clinical symptoms and magnetic resonance angiographic (MRA) results in patients with trigeminal neuralgia and persistent idiopathic facial pain. Medium-term outcome after microvascular decompression of cases with positive MRA findings. Cephalalgia.

[19] Patel NK, Aquilina K, Clarke Y (2003). How accurate is magnetic resonance angiography in predicting neurovascular compression in patients with trigeminal neuralgia? A prospective, single-blinded comparative study. Br J Neurosurg.

[20] Ogiwara M, Shimizu T (2004). Surface rendered three-dimensional MR imaging for the evaluation of trigeminal neuralgia and hemifacial spasm. J Clin Neurosci.

[21] Yamakami I, Kobayashi E, Hirai S (2000). Preoperative assessment of trigeminal neuralgia and hemifacial spasm using constructive interference in steady state-three-dimensional Fourier transformation magnetic resonance imaging. Neurol Med Chir (Tokyo).

[22] Morimoto S, Sasaki S, Miki S (1997). Neurovascular compression of the rostral ventrolateral medulla related to essential hypertension. Hypertension.

[23] Colon GP, Quint DJ, Dickinson LD (1998). Magnetic resonance evaluation of ventrolateral medullary compression in essential hypertension. J Neurosurg.

[24] Watters MR, Burton BS, Turner GE (1996). MR screening for brain stem compression in hypertension. AJNR Am J Neuroradiol.

[25] Levy EI, Scarrow AM, Jannetta PJ (2001). Microvascular decompression in the treatment of hypertension: review and update. Surg Neurol.

[26] Levy EI, Clyde B, McLaughlin MR (1998). Microvascular decompression of the left lateral medulla oblongata for severe refractory neurogenic hypertension. Neurosurgery.

[27] Frank H, Schobel HP, Heusser K (2001). Long-term results after microvascular decompression in essential hypertension. Stroke.

[28] Segal R, Jannetta PJ, Wolfson SK (1982). Implanted pulsatile balloon device for simulation of neurovascular compression syndromes in animals. J Neurosurg.

[29] Morimoto S, Sasaki S, Miki S (2000). Pressor response to pulsatile compression of the rostral ventrolateral medulla mediated by nitric oxide and c-fos expression. Br J Pharmacol.

[30] Morimoto S, Sasaki S, Miki S (1997). Pulsatile compression of the rostral ventrolateral medulla in hypertension. Hypertension.

[31] Bittar RG, Otero S, Carter H (2005). Deep brain stimulation for phantom limb pain. J Clin Neurosci.

[32] Green AL, Owen SL, Davies P (2006). Deep brain stimulation for neuropathic cephalalgia. Cephalalgia.

[33] Owen SL, Green AL, Stein JF (2006). Deep brain stimulation for the alleviation of post-stroke neuropathic pain. Pain.

[34] Richardson DE, Akil H (1977). Long term results of periventricular gray self-stimulation. Neurosurgery.

[35] Bittencourt AS, Carobrez AP, Zamprogno LP (2004). Organization of single components of defensive behaviors within distinct columns of periaqueductal gray matter of the rat: role of N-methyl-D-aspartic acid glutamate receptors. Neuroscience.

[36] Hunsperger RW (1956). [Affective reaction from electric stimulation of brain stem in cats ]. Helv Physiol Pharmacol Acta.

[37] Carrive P (1993). The periaqueductal gray and defensive behavior: functional representation and neuronal organization. Behav Brain Res.

[38] Johnson PL, Lightman SL, Lowry CA (2004). A functional subset of serotonergic neurons in the rat ventrolateral periaqueductal gray implicated in the inhibition of sympathoexcitation and panic. Ann N Y Acad Sci.

[39] Bandler R, Keay KA, Floyd N (2000). Central circuits mediating patterned autonomic activity during active vs. passive emotional coping. Brain Res Bull.

[40] Bandler R, Carrive P, Zhang SP (1991). Integration of somatic and autonomic reactions within the midbrain periaqueductal grey: viscerotopic, somatotopic and functional organization. Prog Brain Res.

[41] Carrive P, Bandler R (1991). Viscerotopic organization of neurons subserving hypotensive reactions within the midbrain periaqueductal grey: a correlative functional and anatomical study. Brain Res.

[42] Carrive P, Bandler R, Dampney RA (1988). Anatomical evidence that hypertension associated with the defence reaction in the cat is mediated by a direct projection from a restricted portion of the midbrain periaqueductal grey to the subretrofacial nucleus of the medulla. Brain Res.

[43] Haselton JR, Guyenet PG (1990). Ascending collaterals of medullary barosensitive neurons and C1 cells in rats. Am J Physiol.

[44] Behbehani MM (1995). Functional characteristics of the midbrain periaqueductal gray. Prog Neurobiol.

[45] Rossi F, Maione S, Berrino L (1994). Periaqueductal gray area and cardiovascular function. Pharmacol Res.

[46] Kabat H, Magoun HW, Ranson SW (1936). Electrical stimulation of points in the forebrain and midbrain. the resultant alterations in blood pressure. J Comp Neurol.

[47] Papanastassiou V, Rowe J, Scott R (1998). Use of the radionics image fusion? and stereoplan? programs for target localization in functional neurosurgery. J Clinical Neuroscience.

[48] Orth RC, Sinha P, Madsen EL (1999). Development of a unique phantom to assess the geometric accuracy of magnetic resonance imaging for stereotactic localization. Neurosurgery.

[49] Joint C, Nandi D, Parkin S (2002). Hardware-related problems of deep brain stimulation. Mov Disord.

[50] Bittar RG, Burn SC, Bain PG (2005). Deep brain stimulation for movement disorders and pain. J Clin Neurosci.

[51] Bendok BR, Levy RM, Onibukon A, Batjer HH, Loftus CM (2003). Deep Brain Stimulation for the Treatment of Intractable Pain. Textbook of neurological surgery: principles and practice.

[52] Young RF, Rinaldi PC, North RB, Levy RM (1997). Brain Stimulation. Neurosurgical Management of Pain.

[53] Green AL, Wang S, Owen SL (2006). Controlling the heart via the brain: a potential new therapy for orthostatic hypotension. Neurosurgery.

[54] Green AL, Wang S, Owen SL (2006). Stimulating the human midbrain to reveal the link between pain and blood pressure. Pain.

[55] Green AL, Wang S, Owen SL (2005). Deep brain stimulation can regulate arterial blood pressure in awake humans. Neuroreport.

[56] Brinton TJ, Cotter B, Kailasam MT (1997). Development and validation of a noninvasive method to determine arterial pressure and vascular compliance. Am J Cardiol.

[57] Mai JK, Assheuer J, Paxinos G (1998). Atlas of the human brain.

[58] Pagani M, Lombardi F, Guzzetti S (1986). Power spectral analysis of heart rate and arterial pressure variabilities as a marker of sympatho-vagal interaction in man and conscious dog. Circ Res.

[59] Pagani M, Montano N, Porta A (1997). Relationship between spectral components of cardiovascular variabilities and direct measures of muscle sympathetic nerve activity in humans. Circulation.

[60] Furlan R, Guzzetti S, Crivellaro W (1990). Continuous 24-hour assessment of the neural regulation of systemic arterial pressure and RR variabilities in ambulant subjects. Circulation.

[61] Task Force of the European Society of Cardiology and the North American Society of Pacing and Electrophysiology (1996). Heart rate variability: standards of measurement, physiological interpretation and clinical use. Circulation.

[62] Lyons KE, Wilkinson SB, Overman J (2004). Surgical and hardware complications of subthalamic stimulation: a series of 160 procedures. Neurology.

[63] Yianni J, Green AL, McIntosh E (2005). The costs and benefits of deep brain stimulation surgery for patients with dystonia: An initial exploration. Neuromodulation.

[64] Phillips NI, Bhakta BB (2000). Affect of deep brain stimulation on limb paresis after stroke. Lancet.

[65] Gotoh TM, Tanaka K, Morita H (2005). Controlling arterial blood pressure using a computer-brain interface. Neuroreport.

[66] Romanelli P, Heit G (2004). Patient-controlled deep brain stimulation can overcome analgesic tolerance. Stereotact Funct Neurosurg.

[67] The Consensus Committee of the American Autonomic Society and the American Academy of Neurology (1996). Consensus statement on the definition of orthostatic hypotension pure autonomic failure and multiple system atrophy.. Neurology.

[68] Kaplan NM (1993). The promises and perils of treating the elderly hypertensive. Am J Med Sci.

[69] Rutan GH, Hermanson B, Bild DE (1992). Orthostatic hypotension in older adults. The Cardiovascular Health Study. CHS Collaborative Research Group. Hypertension.

[70] Inui K, Nosaka S (1993). Target site of inhibition mediated by midbrain periaqueductal gray matter of baroreflex vagal bradycardia. J Neurophysiol.

[71] Cooper VL, Hainsworth R (2001). Carotid baroreceptor reflexes in humans during orthostatic stress. Exp Physiol.

[72] Sanderson JE, Yeung LY, Yeung DT (1996). Impact of changes in respiratory frequency and posture on power spectral analysis of heart rate and systolic blood pressure variability in normal subjects and patients with heart failure. Clin Sci (Lond).

[73] Barbieri R, Bianchi AM, Triedman JK (1997). Model dependency of multivariate autoregressive spectral analysis. IEEE Eng Med Biol Mag.

[74] Nicholas JS, D'Agostino SJ, Patel SJ (2005). Arterial compression of the retro-olivary sulcus of the ventrolateral medulla in essential hypertension and diabetes. Hypertension.

[75] Jannetta PJ, Hollihan L (2004). Type 2 diabetes mellitus, etiology and possible treatment: preliminary report. Surg Neurol.

[76] Fessel J, Robertson D (2006). Orthostatic hypertension: when pressor reflexes overcompensate. Nat Clin Pract Nephrol.

